# Standardizing critical current density measurements in lithium garnets

**DOI:** 10.1038/s42004-023-01002-4

**Published:** 2023-09-09

**Authors:** Matthias Klimpel, Huanyu Zhang, Maksym V. Kovalenko, Kostiantyn V. Kravchyk

**Affiliations:** 1 Laboratory of Inorganic Chemistry, Department of Chemistry and Applied Biosciences, ETH Zürich, 8093 Zürich, Switzerland; 2 Empa - Swiss Federal Laboratories for Materials Science & Technology, 8600 Dübendorf, Switzerland

**Keywords:** Batteries, Batteries

## Abstract

The formation of Li dendrites at the Li/electrolyte interface at practically relevant current densities (> 1 mA cm^−2^) is a critical issue hindering the deployment of non-flammable and non-toxic Li_7_La_3_Zr_2_O_12_ (LLZO) electrolyte in solid-state batteries. In this comment, the authors argue for an agreement to standardize measurements of the critical current density at which Li dendrites begin to penetrate the LLZO solid-state electrolyte.

The quest for high-energy-density Li-ion batteries has led to a surge of reports on various solid-state electrolytes that enable to employ a lithium metal anode^1^. Among the plethora of contenders in the ‘solid-state electrolyte’ domain, Li_7_La_3_Zr_2_O_12_ (LLZO) with the garnet-type structure has attracted considerable attention in recent years due to its high Li-ion conductivity of up to 1 mS cm^−1^ (at room temperature (RT)), low electronic conductivity of ≈ 10^−8^ S cm^−1^ (RT), high thermal stability and chemical compatibility with metallic Li^2–4^.

However, to date, the performance of Li-garnet solid-state batteries (SSB) has not come close to meeting commercial requirements. One of the most prominent issues associated with the use of LLZO for SSBs is the formation of Li dendrites at current densities exceeding 0.3 to 1 mA cm^−2^. According to Ceder et al.^5^, the formation of Li dendrites is caused by the inhomogeneous LLZO surface, which leads to a higher lithium deposition rate in the tip-like regions. As a result, the pressure at the tip of the Li dendrite overcomes the shear modulus of LLZO (149.8 GPa) and consequently penetrates into the solid electrolyte, mainly along the grain boundaries^6,7^. Another closely related problem that triggers Li dendrite formation is the instability of the Li/LLZO interface during cycling. It has been experimentally demonstrated that the formation of voids at the Li/LLZO interface occurs while Li is being stripped^8^. Consequently, this leads to the reduction of the Li/LLZO contact area and the increase of the local current densities at the Li/LLZO interface during the subsequent Li plating. The void formation can therefore induce Li dendrite formation at much lower current densities than those required for dendrite formation in the unstripped Li/LLZO interface.

## Measuring the critical current density and its limitations

Although the formation of Li dendrites in the LLZO solid-state electrolyte is the central issue in this field, there is surprisingly no agreement on the electrochemical protocol required to determine the critical current density (CCD), that is the current density at which Li dendrite propagation begins^9,10^. Currently, it is generally accepted that the CCD can be measured using a symmetric Li/LLZO/Li cell configuration at gradually increasing current densities. The current density at which a sharp potential drop occurs is considered equal to the CCD^11–13^.

However, the limiting conditions for each half-cycle are not standardized^14^, resulting in CCD values for LLZO solid-state electrolyte being different even for identical surfaces^15^. On the one hand, there is the time-limited CCD protocol, in which each half-cycle has a fixed time, most often 30 min. In this case, the amount of plated/stripped Li at the Li/LLZO interface increases with each step as the current density gradually increases. As a result, the difference between the amounts of plated/stripped lithium in the first and last half cycles can be 100 times, often exceeding 1 mAh cm^−2^ (ca. 5 µm in thickness). On the other hand, there is an alternative CCD protocol that uses a fixed capacity limit, e.g. 0.1 mAh cm^−2^. Thus, the same amount of lithium is shuttled in each step, but the time for each step is gradually reduced.

In this comment, we analyze these two most common protocols and their inaccuracies in determining CCDs. In particular, we discuss the critical interplay between the applied boundary condition, e.g., time or areal capacity, and the measured CCD values, considering the effects of void formation at the Li/LLZO interface during CCD measurements. We then propose and experimentally demonstrate an optimized protocol to determine CCD. It should be noted that while temperature^16^ and applied pressure^17,18^, as well as the use of Li/LLZO interfacial layers, and increased Li/LLZO surface area through the employment of LLZO scaffolds^19,20^, can have a significant impact on the obtained CCD values, these contributions are not discussed in this comment.

## Overcoming the limitations

**Fig. 1:**
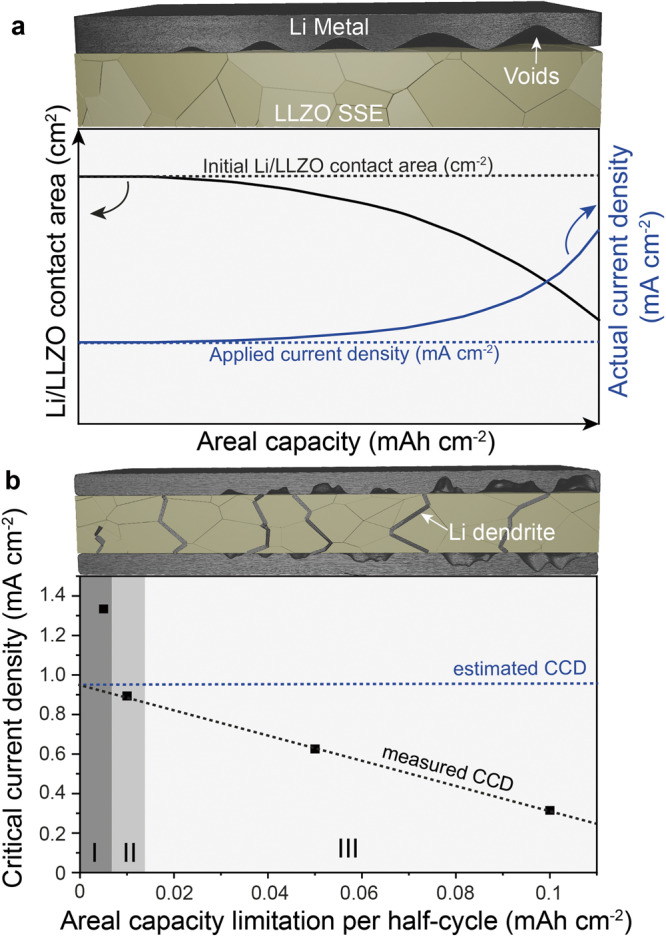
Schematic representation of void and dendrite formation during critical current density (CCD) measurements. **a** Schematics of the impact of the amount of stripped Li from the Li/LLZO interface, shown as areal capacity, on the change of Li/LLZO contact area and actual current density. **b** The measured values of the CCD (black squares) as a function of areal capacity limitation (ACL) per half-cycle. The regions I, II, and III represent the ranges of ACL steps at which CCD values can be considered overestimated, nearly correct and underestimated, respectively. The dashed blue line shows the highest achievable value of CCD (estimated CCD).

In principle, the best approach to determine the CCD is to perform the measurements on multiple full cells comprising high-areal-capacity cathode exhibiting high-rate capability. In this case, the definition of CCD does not refer to any pre-history of the system or the number of cycles but explicitly indicates on the current density required to immediately initiate dendrite formation. The cells should be charged at different current densities without time/areal capacity limitation and the current density at which the particular cell will be shorted can be called the CCD. In this case, the effect of void formation at the Li/LLZO interface on the counter electrode side can be completely eliminated. Considering that such measurements require the use of a cathode, which is often not available, the typical method to determine the CCD is to cycle the symmetrical cells using areal capacity limitation (ACL) step of 0.1 mAh cm^−2^ per half-cycle at gradually increasing current densities. However, a closer look at this protocol shows that the value of the current density obtained from the experiments with symmetric cells does not necessarily correspond to the actual CCD, because the cycling of the symmetric cells concomitantly induces the formation of voids at the Li/LLZO interface and thus reduces the Li/LLZO contact area (Fig. 1a). The latter increases the local current density, inducing the formation of dendrites at current densities that are significantly lower than those anticipated without the void formation (e.g. CCD determined using full cells). In this context, the CCD protocol with time limitation step, e.g., 30 min, would result in even less accurate values, considering that the effect of the void formation would exponentially increase upon increasing the applied current densities. To hinder the formation of voids, Fuchs et al. ^10^ recently suggested to employ pressure and waiting time between the current steps of the measurements, thus closing the previously formed voids during plating/stripping half cycle. Alternatively, we suggest that the optimal approach to measure a CCD value is to minimize the amount of plated/stripped Li per half-cycle at the given current density (the ACL step) and thus the contribution of void formation.

To test our assumptions, namely that the larger the ACL steps lead to the higher contribution of the void formation and thus to lower CCD values, we conducted a series of CCD measurements on symmetrical cells using different ACL steps per half-cycle of 0.1, 0.05, 0.01, and 0.005 mAh cm^−2^ or time limiting step of 30 min. The measurements were performed at 25 °C and a stack pressure of 1.25 MPa. The current density was increased from 0.02 to 3 mA cm^−2^ in steps of 0.02 mA cm^−2^ (Fig. S1 and Supplementary Methods). As can be seen from Fig. 1b, the amount of Li transported per half cycle indeed has a significant contribution to the measured CCD values, which are increasing linearly at lower ACL steps. However, at a low ACL value of 0.005 mAh cm^−2^, the linear dependence breaks down, which can be explained by delayed dendrite formation caused by the amount of plated Li being too small to reach the opposite side of the cell. Therefore, as a rough estimate of the CCD, we suggest to consider taking the current density value obtained by extrapolating the linear part of the current density, as shown on Fig. 1b. Notably, the CCD measurements with time limiting step revealed the lowest value of the current densities of 0.3 mA cm^−2^, which is caused by the severe reduction of the Li/LLZO interface contact area as a result of plating/stripping significantly large amounts of Li compared to the CCD protocol with ACL of 0.01 mA cm^−2^. For example, 30 min at a current density of 0.3 mA cm^−2^ corresponds to approximately 0.75 µm of plated/stripped Li. However, at an ACL of 0.01 mA cm^−2^, only about 0.05 µm of Li is plated/stripped.

## Summary

In summary, our work shows that the protocols of CCD measurements need to be reconsidered, taking into account the issue of void formation at the Li/LLZO interface. Since the latter cannot be eliminated with the symmetrical cell configuration, we suggest performing CCD measurements and therefore reporting CCD values always at different ACL steps of 0.1, 0.05, and 0.01 mAh cm^−2^. Extrapolation of the linear part of the current density function might enable to determine the highest possible achievable CCD. The values obtained at much lower areal capacity limitations might be unrealistic because the ACL step should be long enough to detect a short circuit within a half-cycle when Li dendrite propagation in the LLZO electrolyte starts to progress at a certain current density. Similar considerations apply to CCD measurements of other Li or Na-ion solid-electrolyte systems based on sulfides, halides, Li-ion conducting polymers, or beta-alumina.

### Supplementary information

Supplementary Information

## Data Availability

The data is available from the corresponding authors upon reasonable request.
