# Supplementary material for: Standardizing critical current density measurements in lithium garnets
Source: Commun Chem. 2023 Sep 9;6:192. doi: 10.1038/s42004-023-01002-4 (PMC10492854; doi:10.1038/s42004-023-01002-4)
Supplement: Supplementary file 1 — Supplementary Information [file 42004_2023_1002_MOESM1_ESM.pdf]

Supporting Information for

Standardizing Critical Current Density Measurements in Lithium Garnets

Matthias Klimpel,[1,2] *Huanyu Zhang,*[1,2] *Maksym V. Kovalenko*[1,2*] and *Kostiantyn V. Kravchyk*[1,2*]

[1]Laboratory of Inorganic Chemistry, Department of Chemistry and Applied Biosciences, ETH Zürich, 8093 Zürich, Switzerland

[2]Laboratory for Thin Films and Photovoltaics, Empa - Swiss Federal Laboratories for Materials Science & Technology, 8600 Dübendorf, Switzerland

Corresponding Authors:

*E-mails: mvkovalenko@ethz.ch and Kostiantyn.Kravchyk@empa.ch

Supplementary Methods

240 mg of commercial $Li_{6.25}Al_{0.25}La_3Zr_2O_{12}$ powder (Ampcera, 500 nm nanopowder) was pressed into pellets with a diameter of 10 mm using a uniaxial press at 10 kN force. The surface was cleaned with SiC sand paper and the pellets were dried at 200 °C for 30 min to remove any moisture. Afterwards, they were introduced into an argon filled glovebox (Inert Corp., O_2 < 0.1 ppm, H_2O < 0.1 ppm) and heat-treated at 900 °C for 10 minutes to remove any Li_2CO_3 impurities. The pellets were sintered in a home-built ultra-fast sintering setup with a pre-heating step at 1000 °C for 20 seconds and the sinter step at 1200 °C for 120 seconds. Afterwards, the pellets were treated at 600 °C for two hours in oxygen atmosphere, to remove any carbonaceous impurities on the surface. Finally, the pellets were treated in Argon atmosphere at 900 °C for 10 minutes, again. A fresh piece of lithium from a rod (Sigma Aldrich, 99.9%) was cut and rolled into a foil with a thickness of about 200 µm and punched into electrodes with a diameter of 6 mm. The electrodes were attached with the help of an isostatic press at 1000 kN for three minutes.

The cells were tested inside the argon-filled glovebox with a BioLogic VMP-2 multichannel workstation. To record the critical current density, a current density of 0.02 mA cm^{-2} was applied and increased in steps of 0.02 mA cm^{-2}. Each step was limited to the chosen areal capacity limitation (ACL). The experimental data is shown in Figure S1. Instead of using the time on the x-axis, we chose to use the cumulative areal capacity. As a result, the data is easier to understand, as the width each cycle is the same.

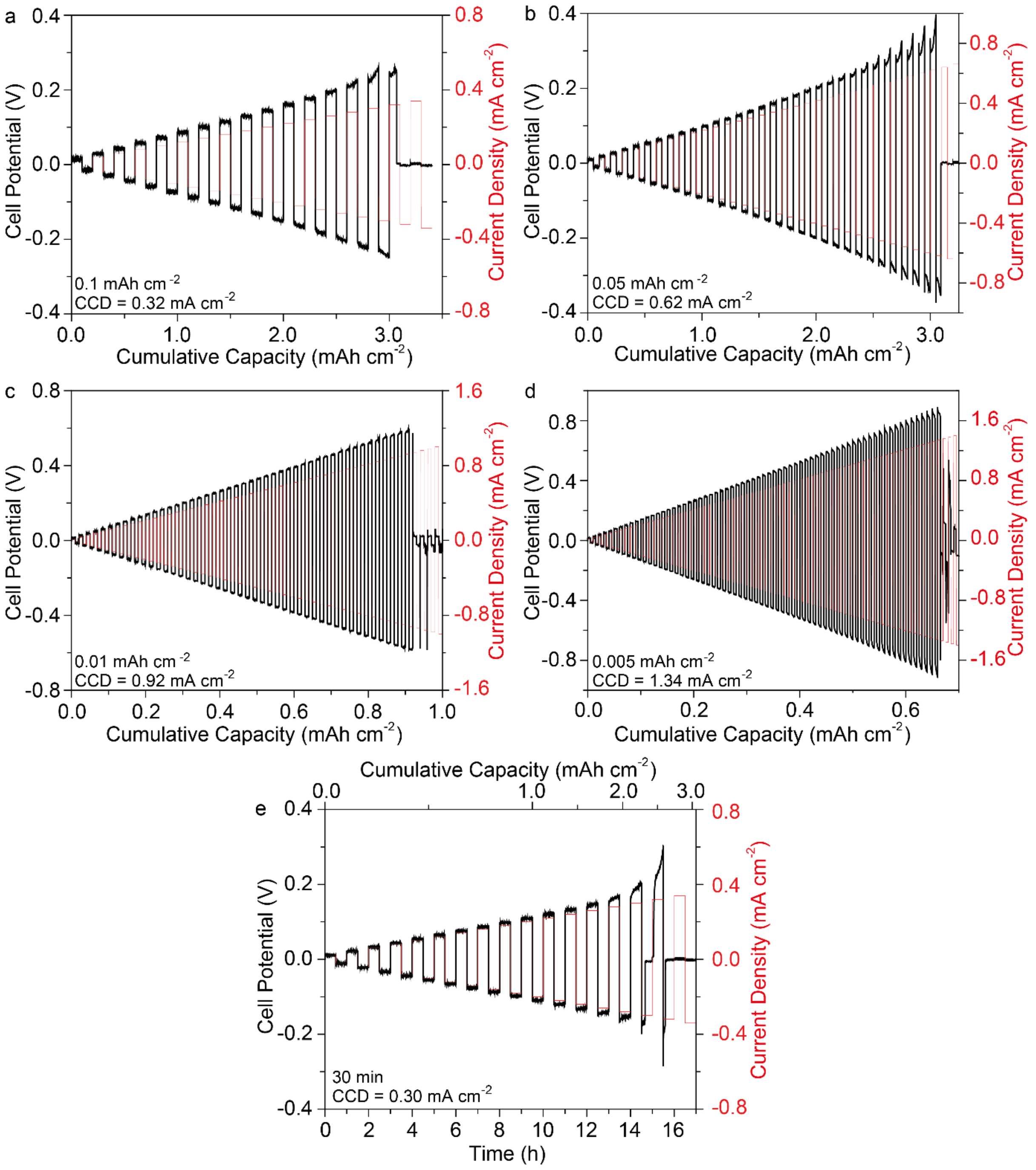

Figure S1. CCD voltage profiles of Li/LLZO/Li symmetrical cells measured with a different capacity/time limitation steps of 0.1 mAh cm^{-2} (a), 0.05 mAh cm^{-2} (b), 0.01 mAh cm^{-2} (c), 0.005 mAh cm^{-2} (d) and 30 min (e) per half-cycle.
